# Rehabilitation Nutrition for Tendon and Ligament Injuries: From Collagen Remodeling to Return-to-Activity and Sport

**DOI:** 10.3390/healthcare14142136

**Published:** 2026-07-16

**Authors:** Eui-Jae Lee, Jooyoung Kim

**Affiliations:** 1 Department of Physical Education, Seowon University, Cheongju 28674, Republic of Korea; pelej@seowon.ac.kr; 2 Department of Health Care Exercise, Seowon University, Cheongju 28674, Republic of Korea

**Keywords:** tendon injury, ligament injury, collagen synthesis, rehabilitation nutrition, energy availability, return-to-activity

## Abstract

**Background/Objectives**: Tendon and ligament injuries are challenging conditions because of limited vascularization, slow collagen turnover, and prolonged rehabilitation timelines. This narrative review aimed to synthesize current evidence on nutritional strategies that may support connective tissue rehabilitation and return-to-activity or return-to-sport progression. **Methods**: This narrative review synthesized the relevant clinical, mechanistic, and applied sports nutrition literature identified through targeted database searches and manual reference screening. **Results**: Adequate energy availability and protein intake emerged as foundational priorities for tissue repair, endocrine and immune function, and tolerance to progressive loading. Collagen or gelatin supplementation with vitamin C before tendon- or ligament-loading exercise may provide targeted substrate and cofactor support for collagen remodeling. Creatine may assist muscle preservation during reduced loading, while micronutrient adequacy and omega-3 fatty acids may support musculoskeletal health and inflammation resolution. **Conclusions**: Nutrition should be viewed as an adjunct to, rather than a replacement for, structured rehabilitation loading. Phase-specific and individualized nutritional strategies may support rehabilitation when integrated with structured loading; however, additional well-designed clinical trials are needed to determine their effects on clinically meaningful outcomes, including return-to-sport time, reinjury risk, and long-term tissue outcomes.

## 1. Introduction

Musculoskeletal injuries remain one of the most common challenges in both elite and recreational sports, accounting for a substantial proportion of time lost from training and competition. Among these injuries, tendon and ligament pathologies—such as anterior cruciate ligament (ACL) rupture, Achilles tendinopathy, and rotator cuff injuries—are particularly problematic due to their slow healing capacity, high reinjury rates, and long rehabilitation timelines [1]. Recovery from connective tissue injury is often measured in months rather than weeks, and incomplete recovery can significantly increase the risk of recurrent injury and long-term functional impairment [2]. Although tendons and ligaments are often discussed together as dense collagen-rich connective tissues, they differ substantially in vascularity, anatomical function, injury mechanisms, surgical requirements, and rehabilitation trajectories. Therefore, the nutritional strategies discussed in this review should be interpreted as broad rehabilitation considerations rather than uniform prescriptions applicable to all tendon and ligament conditions.

Unlike skeletal muscle, tendons and ligaments are characterized by low vascularization, limited cellularity, and slow collagen turnover. These biological constraints contribute to the prolonged remodeling period observed after injury [1,2]. Rehabilitation protocols have traditionally focused on progressive mechanical loading as the primary driver of tissue recovery. However, increasing attention has been directed toward the role of nutritional strategies as modifiable factors that may support extracellular matrix (ECM) remodeling, collagen synthesis, and inflammation resolution during rehabilitation [3].

In recent years, sports nutrition research has made substantial progress in identifying dietary strategies that support skeletal muscle hypertrophy and recovery. Evidence-based guidelines now exist for protein intake, carbohydrate availability, and several ergogenic aids aimed at optimizing muscle repair and adaptation. In contrast, nutritional evidence specifically targeting connective tissue rehabilitation has expanded in recent years, but remains heterogeneous across injury types, study designs, and outcome measures [3]. This gap is noteworthy given that the biological requirements for tendon and ligament repair differ from those of skeletal muscle. While muscle hypertrophy relies heavily on myofibrillar protein synthesis driven by essential amino acids and resistance training, connective tissue repair depends primarily on collagen synthesis, fibroblast activity, and extracellular matrix remodeling [4,5].

Collagen is the predominant structural protein in tendons and ligaments, representing approximately 70–80% of their dry weight [2]. The synthesis and cross-linking of collagen fibers are influenced by several nutritional factors, including protein intake, vitamin C availability, and specific amino acids such as glycine and proline [6]. Furthermore, connective tissue healing is highly sensitive to systemic energy availability and inflammatory status. Energy deficiency, which is common during periods of reduced activity or attempts at body mass management during injury, may impair tissue repair processes and prolong recovery timelines [7]. Emerging evidence also suggests that targeted nutritional strategies—such as collagen or gelatin supplementation combined with vitamin C prior to rehabilitation exercise—may enhance biomarkers of collagen synthesis in response to mechanical loading [8,9].

In addition to macronutrient considerations, several micronutrients and bioactive compounds have been proposed to play supportive roles in connective tissue recovery. Vitamin D and calcium contribute to musculoskeletal health and bone–tendon interface integrity, while omega-3 fatty acids may assist in the resolution of inflammation during later stages of healing [10,11]. Despite growing interest in these approaches, current evidence is dispersed across experimental studies, clinical trials, and applied sports nutrition literature, making it difficult for clinicians and practitioners to translate findings into practical recommendations [3,12].

Several narrative and systematic reviews have addressed nutritional strategies for musculoskeletal injury recovery; however, specific questions remain regarding how energy availability, targeted supplementation, and progressive loading can be integrated within tendon- and ligament-specific rehabilitation contexts. Existing reviews have largely focused on macronutrient strategies for skeletal muscle recovery [3], examined collagen supplementation in isolation from a broader rehabilitation context [12], or addressed musculoskeletal injury broadly without differentiating the distinct biological requirements of tendon and ligament tissues from those of skeletal muscle. Perhaps most notably, the concept of energy availability—and its clinical implications within the Relative Energy Deficiency in Sport (REDs) framework—has received limited attention, specifically in the context of tendon and ligament rehabilitation, despite representing a potentially important and modifiable factor of collagen remodeling and connective tissue recovery [13]. The present review addresses these gaps by: (1) explicitly integrating energy availability and REDs risk as foundational—rather than peripheral—considerations within a connective tissue rehabilitation nutrition framework; (2) positioning the interaction between targeted nutritional strategies and progressive mechanical loading as a mechanistically coherent, clinically applicable approach rather than a list of isolated supplements; and (3) translating the available evidence into a phase-specific framework that aligns nutritional priorities with the biological stages of healing and the evolving demands of return-to-activity or return-to-sport progression.

Therefore, this narrative review aimed to synthesize current evidence on nutritional strategies relevant to tendon and ligament rehabilitation, with particular attention to energy availability, protein intake, collagen-related strategies, creatine, micronutrients, and omega-3 fatty acids. A secondary aim was to distinguish between clinically supported recommendations and mechanistically plausible adjuncts, and to translate the evidence into phase-specific practical considerations for return-to-activity or return-to-sport progression.

## 2. Methods

### 2.1. Review Design

This study was conducted as a narrative review to provide a clinically relevant and mechanistically informed synthesis of nutritional strategies applicable to tendon and ligament rehabilitation. A narrative approach was selected because the current evidence base spans diverse research domains, including clinical rehabilitation studies, sports nutrition research, immobilization models, connective tissue physiology, and mechanistic investigations. These heterogeneous sources of evidence address different aspects of tissue recovery and are not readily amenable to quantitative synthesis. The primary objective of this review was not to estimate pooled treatment effects, but rather to integrate current knowledge regarding energy availability, protein intake, collagen-related strategies, creatine, micronutrients, and omega-3 fatty acids within the context of connective tissue rehabilitation and return-to-activity or return-to-sport progression. Accordingly, this review should be interpreted as an evidence-informed narrative synthesis intended to support clinical decision-making and identify future research priorities. It does not represent a formal systematic review or meta-analysis and was not designed to provide evidence-graded clinical guidelines.

### 2.2. Literature Search Strategy

Relevant literature was identified through targeted searches of PubMed/MEDLINE and Scopus, conducted up to February 2026, focusing primarily on articles published from 2000 onward. This timeframe was selected because most contemporary sports nutrition and rehabilitation research has been published during the past two decades. Additional studies were identified through manual screening of reference lists from relevant review articles, consensus statements, position stands, and key original investigations. Search terms were selected to reflect three broad conceptual domains relevant to tendon and ligament rehabilitation: (1) connective tissue injury and biology (e.g., tendon, ligament, tendinopathy, Achilles tendon, anterior cruciate ligament, ACL, rotator cuff, connective tissue, collagen, extracellular matrix, collagen synthesis, tendon healing, ligament healing); (2) rehabilitation and return-to-activity or return-to-sport progression (e.g., rehabilitation, immobilization, exercise therapy, tendon loading, return-to-activity, return-to-sport); and (3) nutrition-related strategies (e.g., protein, amino acids, collagen, gelatin, vitamin C, creatine, omega-3 fatty acids, energy availability, REDs). The search process was intended to identify literature relevant to the objectives of the review rather than to exhaustively capture all available studies. Therefore, the literature identification strategy should be interpreted as a targeted search process supporting narrative evidence synthesis rather than a formal systematic review procedure.

### 2.3. Eligibility and Evidence Prioritization

Studies were considered eligible if they addressed one or more of the following areas: (1) tendon or ligament injury rehabilitation; (2) connective tissue biology, collagen metabolism, or extracellular matrix remodeling; (3) energy availability, low energy availability (LEA), or Relative Energy Deficiency in Sport (REDs); (4) protein intake, amino acids, collagen or gelatin supplementation, creatine, micronutrients, or omega-3 fatty acids; and (5) nutritional strategies relevant to musculoskeletal rehabilitation, immobilization, return-to-activity, or return-to-sport progression. Given the limited availability of rehabilitation-specific nutrition studies in tendon and ligament populations, evidence from multiple levels of investigation was considered. Clinical studies involving individuals undergoing tendon or ligament rehabilitation were prioritized whenever available. However, mechanistic studies, healthy-participant investigations, immobilization models, sports nutrition studies, and connective tissue physiology research were also included when they provided information relevant to rehabilitation nutrition. Studies were excluded if they focused primarily on unrelated aspects of general nutrition, non-musculoskeletal diseases, or interventions without clear relevance to connective tissue rehabilitation, tissue remodeling, rehabilitation loading, or return-to-activity or return-to-sport progression. Because the objective of this review was to provide a clinically applicable synthesis rather than a formal evidence review, studies were selected based on their relevance to the review objectives, biological rationale, and potential applicability to rehabilitation practice. Only peer-reviewed articles published in English were primarily considered, although key references identified through manual screening were also reviewed when relevant to the scope of the narrative synthesis. Conference abstracts, editorials, letters, and other non-peer-reviewed publications were not considered unless they provided essential background information.

### 2.4. Narrative Synthesis and Evidence Interpretation

**Figure 1:**
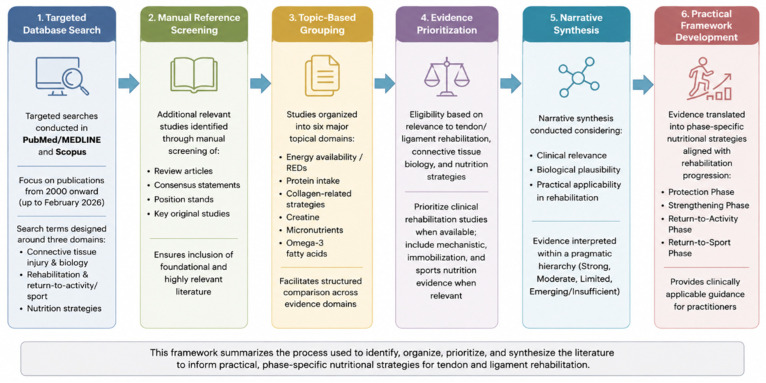
Framework illustrating the targeted literature identification process, evidence prioritization, and narrative synthesis approach used in this review.

The evidence was synthesized narratively according to clinical relevance, biological plausibility, and practical applicability to rehabilitation settings. Because this review integrated evidence from diverse study designs, including clinical rehabilitation studies, randomized controlled trials, observational studies, mechanistic investigations, immobilization models, and applied sports nutrition research, a formal risk-of-bias assessment was not performed. To improve transparency and interpretation, the available evidence was considered within a pragmatic evidence hierarchy. Interventions were interpreted according to the overall strength of supporting evidence rather than on the basis of individual study findings alone. Strong evidence was defined as evidence supported by consensus statements, clinical guidelines, and/or multiple human studies demonstrating consistent findings. Moderate evidence was defined as evidence supported by human studies and biologically plausible mechanisms, but with limited rehabilitation-specific clinical outcomes. Limited evidence was defined as evidence supported primarily by indirect evidence, mechanistic studies, small-sample investigations, or studies conducted outside tendon and ligament rehabilitation populations. Emerging or insufficient evidence was defined as evidence supported mainly by hypothesis-generating data, preliminary findings, or evidence with substantial uncertainty. This framework was used to guide interpretation throughout the review and to distinguish foundational nutritional priorities from adjunctive or emerging strategies. The overall process of literature identification, evidence prioritization, and narrative synthesis is illustrated in Figure 1. The evidence hierarchy is summarized in Table 1.

## 3. Biology of Tendon and Ligament Healing

Tendons and ligaments are composed primarily of type I collagen (~70–80% dry weight), arranged in hierarchical fibrillar structures that provide tensile strength and stiffness [14]. Both tissues are characterized by low cellularity, poor vascularization, and slow collagen turnover—biological constraints that limit intrinsic healing capacity and prolong recovery timelines [15].

Healing proceeds through three overlapping phases: an inflammatory phase lasting days, during which immune cells clear damaged matrix and initiate repair; a proliferative phase lasting weeks, characterized by fibroblast activity and rapid type III collagen synthesis; and a remodeling phase lasting months, during which type III collagen is gradually replaced by stronger type I collagen aligned along loading directions [16]. These processes depend on sufficient amino acid availability, vitamin C, and adequate energy intake, particularly during collagen synthesis and maturation.

Functional recovery therefore depends not only on symptom resolution but also on restoration of collagen organization, cross-link maturation, and mechanical stiffness. Because collagen cross-linking requires vitamin C as an essential enzymatic cofactor, recovery should be conceptualized in terms of both collagen quantity and quality [6,8]. Importantly, nutrition and mechanical loading may interact during remodeling; providing targeted nutritional substrates around loading sessions may amplify collagen synthesis responses [8]. This interaction forms the mechanistic basis for the phase-specific strategies outlined in this review.

Although tendons and ligaments differ in vascularity and healing trajectories, for example, ACL rupture often requires surgical reconstruction because of limited intrinsic healing capacity, both tissues share fundamental nutritional requirements for collagen remodeling and ECM repair [13].

Connective tissue healing should not be reduced solely to collagen synthesis. Functional recovery also depends on matrix organization, cross-link maturation, neuromuscular control, pain modulation, vascular and cellular responses, and progressive restoration of load tolerance.

## 4. Energy Availability and Tissue Healing

Energy availability (EA)—defined as dietary energy intake minus exercise energy expenditure, normalized to fat-free mass—is a foundational determinant of recovery during tendon and ligament rehabilitation. Although training volume often decreases after injury, tissue repair is not a passive process [17]. Immune activity, protein turnover, collagen synthesis, angiogenesis, neuromuscular retraining, and progressive rehabilitation exercise all require sustained metabolic support. Therefore, individuals undergoing rehabilitation may remain metabolically vulnerable even when overall physical activity or sport participation is reduced [17].

Low energy availability (LEA) may be particularly common during rehabilitation because individuals may reduce food intake in response to lower training volume, appetite suppression, pain, psychological stress, or concerns about body composition. This risk may be amplified in weight-sensitive or aesthetic activities and in individuals who add cross-training to maintain fitness while unintentionally failing to match increased energy expenditure. When energy intake is insufficient, amino acids may be diverted toward energy production rather than tissue repair, reducing the efficiency of protein- and collagen-based strategies [7,17].

From a connective tissue perspective, energy deficiency may compromise collagen synthesis and extracellular matrix remodeling by limiting ATP availability, substrate supply, and the endocrine environment required for repair [8]. LEA is associated with alterations in anabolic hormones, thyroid function, immune status, and, in physically active females, menstrual function and sex hormone regulation [18]. These changes may impair collagen metabolism, prolong inflammation, and reduce tolerance to progressive loading. Clinically, this is important because symptoms may improve before structural tissue integrity and load tolerance are fully restored, potentially contributing to increased susceptibility to reinjury during return-to-activity or return-to-sport progression, although direct clinical evidence remains limited.

The REDs framework is therefore relevant to rehabilitation nutrition, particularly in physically active individuals and sports participants. Individuals presenting with recent weight loss, restrictive eating, low carbohydrate availability, menstrual irregularities, persistent fatigue, frequent illness, poor sleep, or stalled rehabilitation progress should be screened for LEA/REDs risk. In these cases, restoring adequate energy intake should take priority before implementing targeted supplementation strategies [7].

In practical terms, intentional energy restriction should generally be avoided during the early stages of tendon and ligament rehabilitation [3]. Rather than aggressively reducing total intake because training volume has decreased, individuals should maintain regular meals and adjust intake according to rehabilitation load, body composition goals, and recovery status. As loading progresses, energy and carbohydrate availability should increase to support high-quality rehabilitation sessions, particularly tendon-loading, strength, neuromuscular, and sport-specific training [19]. Adequate carbohydrate intake is especially important because it supports training quality and may indirectly provide a supportive environment for connective tissue remodeling by enabling more effective mechanical loading. Thus, energy sufficiency should be considered a foundational nutritional priority that may help support collagen remodeling and tolerance to progressive rehabilitation loading [3].

## 5. Protein Intake and Amino Acids

Protein intake is a foundational component of rehabilitation nutrition because it supports both skeletal muscle preservation and connective tissue repair [20]. During tendon and ligament rehabilitation, individuals must maintain muscle mass and strength despite reduced loading, while also providing amino acid substrates required for collagen synthesis, extracellular matrix remodeling, and tissue maturation [20,21]. However, connective tissue recovery should not be viewed simply as an extension of muscle hypertrophy. Whereas skeletal muscle adaptation is strongly influenced by essential amino acids and leucine-mediated myofibrillar protein synthesis [22], tendon and ligament repair depend more heavily on collagen deposition, collagen maturation, cross-link formation, and structural reorganization in response to progressive mechanical loading [21].

Individuals recovering from tendon or ligament injury may require higher protein intake because of inflammation-related catabolism, immobilization-induced anabolic resistance, and the ongoing demands of tissue repair [3,20]. In applied rehabilitation settings, daily protein intakes of approximately 1.6–2.2 g/kg/day are commonly recommended to support muscle protein synthesis, limit disuse-related muscle loss, and sustain whole-body recovery [22,23]. These recommendations are derived primarily from the broader sports nutrition and skeletal muscle literature rather than tendon- or ligament-specific rehabilitation trials, reflecting the limited availability of direct connective tissue evidence. For connective tissue repair, adequate protein also helps maintain the amino acid pool required for collagen production, particularly glycine, proline, and hydroxyproline [8,21]. Importantly, protein strategies are most effective when energy availability is sufficient; under conditions of low energy availability, amino acids may be oxidized for energy rather than directed toward repair and remodeling [3].

Protein distribution across the day is also relevant during rehabilitation. Providing repeated protein doses, typically around 25–40 g per meal or approximately 0.3–0.4 g/kg/meal, can help maintain net protein balance and provide regular anabolic support during periods of reduced activity [24,25]. Although this approach is often discussed in relation to skeletal muscle, it may also support connective tissue recovery by ensuring consistent substrate availability during the prolonged remodeling process [21]. This is especially important when appetite, meal timing, or dietary routine is disrupted after injury [3].

Protein quality should be considered in relation to the dual goals of rehabilitation. Leucine-rich, high-quality proteins such as whey, dairy, eggs, fish, and lean meats are useful for supporting muscle preservation and strength recovery [22]. In contrast, collagen-rich proteins, gelatin, or collagen peptides provide higher amounts of glycine and proline, which are more directly relevant to collagen synthesis [8]. Therefore, a practical strategy is not to replace high-quality dietary protein with collagen, but to combine general protein adequacy with targeted collagen-specific approaches when appropriate. This distinction is important because collagen supplementation alone does not provide a complete amino acid profile for muscle maintenance or whole-body recovery [8,22].

Timing may also be important, particularly when protein strategies are aligned with rehabilitation loading. General high-quality protein intake can be distributed across meals and after rehabilitation sessions to support muscle recovery [22], while collagen- or gelatin-based strategies may be most relevant before key tendon- or ligament-loading sessions [8]. These may include heavy, slow resistance exercise, eccentric loading, progressive isometrics, or plyometric reintroduction. In this context, protein intake functions as part of a broader rehabilitation nutrition strategy: high-quality protein supports muscle and systemic recovery, whereas collagen-specific amino acid provision may support extracellular matrix remodeling when paired with appropriate mechanical loading [8,21].

In practical terms, individuals recovering from tendon or ligament injury should prioritize consistent daily protein intake, regular meal distribution, and adequate total energy intake before relying on isolated supplementation. Liquid protein options, protein-enriched snacks, and pre-sleep protein may be useful when appetite or routine is disrupted, but these should be integrated into a complete dietary plan [26]. Overall, protein intake during tendon and ligament rehabilitation should be framed as a dual-purpose strategy: preserving the muscular capacity needed for progressive loading while potentially supporting collagen remodeling during rehabilitation and progression toward return-to-activity or return-to-sport.

## 6. Muscle Preservation During Rehabilitation: The Role of Creatine

Although creatine does not directly target collagen synthesis or tendon–ligament remodeling, it may have functional relevance during rehabilitation by supporting skeletal muscle preservation and training capacity [27]. This distinction is important because progressive mechanical loading remains the primary stimulus for connective tissue adaptation, and effective loading depends on the strength and fatigue resistance of the surrounding musculature [21]. After tendon or ligament injury, immobilization, reduced activity, pain, and altered movement patterns can accelerate muscle loss and strength deficits, which may limit rehabilitation progression and compromise return-to-activity or return-to-sport [20].

Creatine monohydrate may help attenuate disuse-related declines in muscle mass and strength by increasing intramuscular phosphocreatine availability and supporting ATP resynthesis during repeated or high-intensity contractions [27]. These effects may be particularly relevant as rehabilitation progresses from early isometric or low-load exercise toward resistance training, plyometric reintroduction, and sport-specific loading. In this context, creatine should be viewed as a muscle-supportive strategy that may help support the individual’s capacity to perform high-quality rehabilitation work, rather than as a connective tissue-specific intervention [21,27].

Evidence from immobilization and rehabilitation-related models suggests that creatine supplementation may support lean mass, strength recovery, and functional performance when combined with structured exercise [28]. However, direct evidence in individuals undergoing tendon or ligament rehabilitation remains limited, and not all immobilization studies show a protective effect during short-term disuse [29]. Therefore, its use should be interpreted cautiously and positioned as an adjunct to foundational strategies such as adequate energy availability, sufficient protein intake, and progressive rehabilitation loading.

In practice, creatine monohydrate may be considered when the primary concern is disuse-related muscle loss, prolonged immobilization, or difficulty restoring strength during rehabilitation. A practical approach is 3–5 g/day, with or without a short loading phase, depending on urgency, tolerance, and practitioner preference [27]. Creatine should be integrated within a complete rehabilitation nutrition plan and should not replace energy, protein, collagen-specific strategies, or individualized loading progression.

## 7. Collagen or Gelatin Supplementation Combined with Vitamin C

Collagen or gelatin supplementation combined with vitamin C is one of the most relevant targeted nutritional strategies for tendon and ligament rehabilitation [8,30]. Unlike skeletal muscle, which adapts primarily through myofibrillar protein synthesis, tendon and ligament recovery depend on collagen synthesis, extracellular matrix remodeling, fibril alignment, and gradual restoration of mechanical stiffness [31,32]. These processes are strongly influenced by progressive mechanical loading, but they also require sufficient substrate availability and micronutrient support. Therefore, collagen-focused supplementation may be considered a practical adjunct to foundational strategies such as adequate energy availability and total protein intake [13,30].

The rationale for collagen or gelatin supplementation is based on the amino acid composition of connective tissue. Collagen is rich in glycine, proline, and hydroxyproline, which are required for collagen triple helix formation, structural stability, and extracellular matrix integrity [33]. Although some of these amino acids can be synthesized endogenously, the demand for collagen turnover may increase during injury repair and progressive rehabilitation. Traditional high-quality proteins such as whey, casein, eggs, fish, and lean meats remain essential for whole-body recovery and muscle preservation, but they provide relatively lower amounts of collagen-specific amino acids compared with gelatin or collagen peptides [22,33]. Thus, collagen supplementation should be viewed as a targeted substrate strategy rather than a replacement for complete dietary protein.

Vitamin C is central to this strategy because it acts as a cofactor for prolyl and lysyl hydroxylase enzymes involved in collagen synthesis [6]. These hydroxylation reactions stabilize the collagen triple helix and support cross-link formation, both of which are important for tensile strength and tissue maturation [34]. Although severe vitamin C deficiency is uncommon in athletes, suboptimal intake may occur during periods of reduced appetite, restricted dietary variety, travel, or intentional energy restriction after injury. Combining collagen or gelatin with vitamin C therefore provides both the amino acid substrates and enzymatic support required for collagen formation and maturation [8,33].

The timing of collagen intake relative to rehabilitation loading is particularly important. Mechanical loading provides the primary adaptive signal for tendon and ligament remodeling, whereas collagen-derived amino acids and vitamin C may support the biochemical environment in which remodeling occurs [31,33]. Ingesting collagen or gelatin before loading exercise may increase circulating collagen-derived amino acids during the period when connective tissues are exposed to mechanical stress [8,30]. This strategy is most relevant before rehabilitation sessions that provide meaningful tendon or ligament loading, such as heavy slow resistance exercise, eccentric loading, progressive isometrics, or plyometric reintroduction.

In practical terms, commonly used protocols involve approximately 10–15 g of gelatin or hydrolyzed collagen combined with vitamin C, ingested about 30–60 min before key rehabilitation loading sessions [8,30]. Hydrolyzed collagen may be preferred in practice because of its solubility and ease of use, whereas gelatin can also be used depending on availability and tolerance [30]. This strategy should be implemented selectively around high-value rehabilitation sessions rather than applied indiscriminately to all activities. It should also be integrated only after foundational energy and protein needs are being met, because collagen supplementation is unlikely to compensate for low energy availability, inadequate total protein intake, or poorly structured loading progression [33].

Despite its practical appeal, the current evidence should be interpreted cautiously. Much of the literature is based on small human studies, short-term interventions, or surrogate markers of collagen synthesis and connective tissue turnover rather than long-term clinical endpoints [33]. Evidence directly linking collagen supplementation to faster return-to-activity or return-to-sport, lower reinjury risk, improved tissue mechanics, or superior functional outcomes in individuals undergoing rehabilitation remains limited [33]. Heterogeneity in injury type, tissue site, rehabilitation design, supplement form, dose, and timing further limits generalizability. Therefore, collagen or gelatin with vitamin C should be presented as a promising, mechanistically plausible adjunct—not a standalone treatment or guaranteed accelerator of healing.

Overall, collagen or gelatin supplementation combined with vitamin C may be considered a potentially useful adjunct when paired with structured tendon- or ligament-loading exercise during rehabilitation. Its role is best interpreted as providing substrate and cofactor support for collagen remodeling in conjunction with appropriate loading, rather than as an intervention proven to accelerate return-to-sport or reduce reinjury risk.

## 8. Micronutrients in Connective Tissue Recovery

Micronutrient sufficiency is an important supportive factor during tendon and ligament rehabilitation because several micronutrients contribute to collagen maturation, enzymatic cross-link formation, immune regulation, and bone–tendon interface integrity [13,35]. Even when energy and protein intake are adequate, insufficient micronutrient availability may limit the biological processes required for extracellular matrix remodeling and adaptation to progressive loading [35]. This is particularly relevant during injury rehabilitation, when individuals may reduce total food intake, narrow dietary variety, or rely more heavily on convenience foods despite ongoing repair demands [3].

Vitamin C is especially relevant because it supports collagen hydroxylation and cross-link formation, as discussed in the collagen supplementation section [6,34]. Beyond its role when combined with collagen or gelatin, adequate vitamin C status should be maintained throughout rehabilitation because collagen maturation and remodeling continue beyond the early healing phase [36]. In practice, this can be supported through regular intake of vitamin C-rich foods, with modest supplementation considered when intake is low or dietary variety is limited.

Vitamin D and calcium are also important within the broader musculoskeletal recovery framework. Vitamin D status may influence immune function, muscle function, bone metabolism, and potentially connective tissue remodeling pathways [37]. Deficiency or insufficiency may be more common in individuals with limited sun exposure, indoor training, or winter-season rehabilitation; therefore, vitamin D should be managed using a status-driven approach, with assessment and individualized correction when feasible [37]. Calcium supports bone remodeling and mineralization, which is particularly relevant at the enthesis, where tendon or ligament attaches to bone and where force transfer increases during late rehabilitation and return-to-activity or return-to-sport progression [13].

Micronutrient adequacy is closely linked to energy availability. Individuals who intentionally reduce energy intake during injury downtime may also reduce intake of fruits, vegetables, dairy products, fortified alternatives, and other nutrient-dense foods [7]. This can increase the risk of suboptimal vitamin C, vitamin D, calcium, and broader micronutrient status, potentially compromising rehabilitation progress and tolerance to progressive loading [13]. A food-first approach should therefore be prioritized, with targeted supplementation used selectively when dietary intake is insufficient, deficiency is confirmed, or risk factors such as low energy availability, restrictive eating, indoor training, winter season, or previous bone stress injury are present [37,38].

Overall, micronutrients should be positioned as supportive enablers of connective tissue recovery rather than standalone interventions. Ensuring adequate vitamin C, vitamin D, and calcium intake may help support collagen maturation, immune function, bone–tendon interface health, and the individual’s ability to progress safely toward higher loading demands during rehabilitation.

## 9. Omega-3 Fatty Acids and Inflammation Resolution

Inflammation is a necessary component of tendon and ligament healing. In the early phase after injury, inflammatory signaling helps clear damaged extracellular matrix, recruit immune and reparative cells, and initiate downstream processes involved in collagen synthesis and tissue remodeling [39,40]. Therefore, the goal of nutritional support should not be to aggressively suppress inflammation. Instead, a more appropriate framework is to support a timely transition from inflammation toward repair and remodeling [40].

Omega-3 fatty acids, particularly eicosapentaenoic acid and docosahexaenoic acid, may contribute to this process by serving as precursors for specialized pro-resolving mediators such as resolvins and protectins [41]. These lipid mediators are involved in regulating immune activity and promoting resolution of inflammation [41,42]. In this context, omega-3 fatty acids should be viewed less as nonspecific anti-inflammatory agents and more as nutritional substrates that may support inflammation resolution and recovery environment optimization [42].

The potential relevance of omega-3 fatty acids during tendon and ligament rehabilitation is also indirect. EPA and DHA may influence inflammatory signaling, membrane lipid composition, muscle protein metabolism, and possibly fibroblast activity or extracellular matrix turnover [42,43]. Their possible role in attenuating muscle loss during periods of reduced loading is clinically relevant because strength deficits and neuromuscular deconditioning can impair rehabilitation progression and reduce tolerance to mechanical loading [44,45]. Thus, omega-3 intake may contribute to a favorable rehabilitation environment by supporting both inflammation resolution and preservation of the muscular capacity needed for progressive loading.

In practice, omega-3 intake should be integrated into a broader rehabilitation nutrition strategy rather than treated as a standalone intervention. Dietary sources such as fatty fish may be encouraged, and supplementation can be considered when habitual intake is low. Common practical strategies use approximately 2–3 g/day of combined EPA and DHA, although dosing should be interpreted carefully because total fish oil content is not equivalent to EPA + DHA content [38,42]. Omega-3 supplementation should be implemented alongside adequate energy availability, sufficient protein intake, and structured rehabilitation loading.

Current evidence remains limited with respect to direct tendon- or ligament-specific clinical outcomes. Most available studies focus on inflammatory markers, muscle preservation, or general musculoskeletal recovery rather than return-to-activity or return-to-sport time, reinjury risk, tissue mechanics, or imaging-based tendon and ligament quality [45]. Therefore, omega-3 fatty acids should be presented as a plausible supportive adjunct, not as a proven intervention for accelerating connective tissue healing. Their most appropriate role may be during the transition from early healing to progressive loading, when inflammation resolution, muscle preservation, and training tolerance become central to rehabilitation progression.

## 10. Practical Considerations for Rehabilitation and Return-to-Activity or Return-to-Sport

The following considerations should not be interpreted as formal evidence-graded clinical guidelines, but as practical strategies derived from current evidence, mechanistic rationale, and applied sports nutrition practice. The preceding sections indicate that nutrition should be integrated into tendon and ligament rehabilitation as a supportive strategy rather than an isolated treatment [3,13]. In practice, the goal is to create a physiological environment that may support collagen remodeling, preserve muscle function, improve tolerance to progressive loading, and facilitate a progression toward return-to-activity or return-to-sport [21]. Therefore, nutritional planning should be coordinated with the rehabilitation phase, loading progression, injury type, and individual risk factors.

The first priority is to ensure adequate energy availability and protein intake. Intentional energy restriction should generally be avoided during early rehabilitation because low energy availability may impair collagen remodeling, endocrine function, immune function, and tolerance to progressive loading [7]. Protein intake should remain consistently high, commonly around 1.6–2.2 g/kg/day, with regular distribution across meals to support muscle preservation and tissue repair [23,25]. Carbohydrate intake should also be adjusted according to rehabilitation load, particularly when strength training, neuromuscular work, or sport-specific conditioning increases [19].

Targeted supplementation may then be considered after foundational needs are met. Collagen or gelatin combined with vitamin C is most relevant before key tendon- or ligament-loading sessions, such as heavy slow resistance exercise, eccentric loading, progressive isometrics, or plyometric reintroduction [8,30]. A practical strategy is approximately 10–15 g of collagen or gelatin with vitamin C, ingested about 30–60 min before these sessions [8,33]. Creatine monohydrate may be considered when immobilization or reduced loading increases the risk of muscle loss, with 3–5 g/day serving as a practical maintenance dose [27]. Omega-3 fatty acids may be used as an adjunct to support inflammation resolution and the recovery environment, particularly when dietary intake of fatty fish is low; a commonly used target is approximately 2–3 g/day of combined EPA and DHA [38,42].

Micronutrient adequacy should be assessed throughout rehabilitation, especially in individuals with low energy availability, restricted diets, indoor training, winter-season rehabilitation, or a history of bone stress injury [7,37]. Vitamin C supports collagen maturation, vitamin D supports musculoskeletal and immune function, and calcium contributes to bone remodeling and enthesis integrity [13]. A food-first approach should be prioritized, with supplementation used selectively when intake is inadequate or a deficiency is identified [38].

As summarized in Table 2, nutritional strategies should evolve across rehabilitation phases. In the early phase, the emphasis should be on energy sufficiency, adequate protein, micronutrient adequacy, and prevention of excessive muscle loss. During the strengthening and reconditioning phase, nutrition should support higher-quality loading sessions, muscle rebuilding, and collagen remodeling. During the late return-to-activity or return-to-sport phase, energy and carbohydrate intake should increase in parallel with training demands, while collagen timing, omega-3 intake, and micronutrient correction may be maintained when clinically relevant [3,13].

Finally, rehabilitation nutrition should be individualized and coordinated within the multidisciplinary team. Injury type, surgical status, rehabilitation load, body composition goals, sex-specific considerations, dietary preferences, gastrointestinal tolerance, and supplement acceptability should all guide implementation [46]. Nutrition should not replace progressive loading or return-to-activity or return-to-sport criteria, but it may help support the individual’s capacity to perform rehabilitation, may help support tissue remodeling, and may help reduce barriers to a safe and durable return-to-activity or return-to-sport [47].

## 11. Future Research Directions

Despite increasing interest in rehabilitation nutrition, the evidence base for nutritional strategies targeting tendon and ligament healing remains relatively limited compared with the extensive literature on skeletal muscle adaptation [33]. Much of the current knowledge is derived from mechanistic studies, short-term human trials, and indirect evidence from muscle physiology or general injury recovery research [33]. As a result, several important research gaps remain, particularly regarding translation from biomarker endpoints to clinically meaningful outcomes and the integration of nutrition with rehabilitation loading progression.

First, more long-term randomized controlled trials are needed to evaluate whether nutritional interventions can meaningfully improve clinical rehabilitation outcomes such as return-to-activity or return-to-sport timing, reinjury rates, long-term tissue function, and objective performance metrics [33,47]. Although acute changes in collagen synthesis markers, inflammation resolution mediators, or muscle protein synthesis outcomes are valuable for mechanistic insight, their translation into meaningful functional outcomes remains uncertain and is a key research priority. Future trials should incorporate standardized rehabilitation protocols, clear return-to-activity or return-to-sport criteria, and longer follow-up periods to capture reinjury risk and tissue durability beyond short-term symptom improvement [47,48,49].

Second, the optimal dosing, timing, and duration of collagen or gelatin supplementation require further investigation. Current recommendations are based on a relatively small number of studies, and it remains unclear whether different injury types (acute rupture, chronic tendinopathy, post-surgical reconstruction), rehabilitation stages, or study populations require distinct protocols [30,33]. In addition, future studies should clarify whether collagen supplementation provides additive benefits beyond high total protein intake and adequate energy availability, and whether responder characteristics (baseline diet quality, age, sex, connective tissue phenotype) influence efficacy.

Third, research on females, including physically active women and athletes, remains underrepresented. Sex-specific considerations—including hormonal fluctuations, differences in collagen metabolism, menstrual function, LEA/REDs risk, and higher prevalence of certain ligament injuries such as ACL rupture—suggest that further research is needed to better understand nutritional requirements and timing strategies during rehabilitation [7,18,31]. Future work should also examine the interaction between menstrual function, LEA/REDs risk, and connective tissue healing outcomes, as these factors may influence both tissue remodeling and reinjury risk during return-to-activity or return-to-sport progression.

Fourth, the interaction between energy availability, REDs, and injury recovery warrants further study. While the negative effects of low energy availability on performance and health are well documented, its direct impact on connective tissue healing, collagen remodeling quality, enthesis recovery, and reinjury risk remains insufficiently explored [7,31]. Future research should evaluate whether improving energy availability during rehabilitation influences tissue recovery, improves tolerance to progressive loading, and may influence reinjury risk, and should clarify practical screening and intervention approaches for LEA/REDs risk during injury downtime.

Finally, future research should investigate the combined effects of nutrition and rehabilitation loading protocols. Rehabilitation outcomes are strongly influenced by progressive loading design, strength reconditioning, plyometric exposure, and sport-specific demands. Therefore, nutrition trials should be designed with loading progression as a central component rather than a background variable [50,51,52]. Studies that integrate nutrition strategies with progressive resistance training, tendon-loading frameworks, plyometrics, and return-to-activity or return-to-sport programs—while measuring both clinical outcomes and mechanistic endpoints—will be essential to refine evidence-based guidelines and strengthen the integration of nutrition into tendon and ligament rehabilitation.

## 12. Strengths and Limitations

A strength of this review is that it integrates evidence from sports nutrition, connective tissue biology, immobilization models, and clinical rehabilitation to provide a clinically oriented framework for tendon and ligament rehabilitation. The review also emphasizes energy availability and REDs as underrecognized considerations and distinguishes foundational nutritional priorities from adjunctive strategies with varying levels of supporting evidence.

Several limitations should be acknowledged. First, this review used a narrative rather than a systematic methodology; therefore, study selection may be subject to selection bias, and no formal risk-of-bias assessment was performed. Second, the evidence base includes heterogeneous study designs, including mechanistic, indirect, and small-sample studies, limiting the strength of clinical recommendations. Third, tendon and ligament injuries differ by tissue type, injury mechanism, surgical status, and rehabilitation pathway; therefore, the strategies discussed should be interpreted as broad evidence-informed considerations rather than uniform prescriptions.

## 13. Conclusions

Tendon and ligament injuries require prolonged rehabilitation because connective tissue recovery depends on gradual extracellular matrix remodeling, restoration of tissue organization, and progressive recovery of load tolerance. Progressive mechanical loading remains the primary stimulus for adaptation, but nutrition may help provide the systemic and nutritional environment necessary to support effective tissue repair.

This narrative review highlights that adequate energy availability and protein intake should be prioritized before targeted supplementation strategies are considered. Collagen or gelatin combined with vitamin C may support collagen remodeling when paired with tendon- or ligament-loading exercise, while micronutrient adequacy, omega-3 fatty acids, and creatine may provide additional support in selected rehabilitation contexts, particularly for muscle preservation and musculoskeletal recovery during periods of reduced loading.

Overall, nutritional management should be considered a phase-specific and individualized component of multidisciplinary rehabilitation. Importantly, nutrition should complement—not replace—structured rehabilitation loading. Current evidence most strongly supports adequate energy availability and protein intake as foundational nutritional strategies, while collagen or gelatin supplementation with vitamin C, creatine, micronutrient correction, and omega-3 fatty acids may provide additional support in selected rehabilitation contexts. However, these interventions are supported by varying levels of evidence, and further well-designed randomized controlled trials are required to determine their effects on clinically meaningful outcomes, including functional recovery, return-to-activity or return-to-sport timing, reinjury risk, and long-term tissue outcomes.

## Figures and Tables

**Table 1:** Summary of nutritional strategies, proposed mechanisms, practical considerations, and level of supporting evidence.

Nutritional Strategy	Proposed Mechanisms Relevant to Tendon/Ligament Rehabilitation	Practical Considerations	Level of Supporting Evidence ^a^	Interpretation
Energy availability (REDs prevention) ^b^	Supports endocrine function, immune competence, collagen remodeling, tissue repair, and tolerance to rehabilitation loading.	Maintain adequate energy intake; assess and correct LEA/REDs risk throughout rehabilitation.	Strong	Foundational priority for connective tissue rehabilitation.
Protein intake	Provides amino acids for collagen synthesis and muscle protein turnover; supports preservation of muscle mass and function during rehabilitation.	Approximately 1.6–2.2 g·kg^−1^·day^−1^, distributed evenly across meals according to individual needs.	Strong	Foundational priority for rehabilitation and recovery.
Collagen or gelatin + Vitamin C	Provides collagen-specific amino acids (e.g., glycine and proline) together with vitamin C as a cofactor; may enhance collagen synthesis in response to mechanical loading.	Approximately 10–15 g collagen/gelatin with 50–100 mg vitamin C consumed 30–60 min before tendon- or ligament-loading exercise.	Moderate	Promising adjunct supported by mechanistic evidence and limited clinical studies.
Creatine	Supports preservation of muscle mass and strength during reduced loading; may improve rehabilitation tolerance.	Approximately 3–5 g creatine monohydrate daily; loading phase optional.	Limited	Muscle-supportive adjunct with limited direct evidence in tendon and ligament rehabilitation.
Vitamin D	Supports musculoskeletal health, collagen synthesis, and osteotendinous integrity, particularly in deficient individuals.	Assess vitamin D status and correct deficiency; maintain serum 25(OH)D concentrations ≥30 ng/mL where appropriate.	Limited–Moderate	Address deficiency when present; supportive rather than primary intervention.
Calcium	Supports bone–tendon interface health and normal musculoskeletal function.	Approximately 1000–1200 mg·day^−1^ from dietary sources and/or supplementation if intake is inadequate.	Limited–Moderate	Ensure adequate intake, particularly during bone or ligament healing.
Omega-3 fatty acids (EPA + DHA)	May support inflammation resolution, muscle protein synthesis, and attenuation of exercise-induced muscle damage.	Approximately 2–3 g EPA + DHA daily when dietary intake is insufficient.	Limited	Potential adjunct supported primarily by mechanistic and indirect evidence.

Note: ^a^ Evidence levels were assigned based on the overall strength and consistency of the available literature. Strong evidence indicates support from consensus statements, clinical guidelines, and/or multiple human studies with consistent findings; Moderate evidence indicates support from human studies and biologically plausible mechanisms but limited rehabilitation-specific clinical outcomes; Limited evidence indicates support mainly from indirect evidence, mechanistic studies, small-sample investigations, or studies outside tendon and ligament rehabilitation populations; Emerging/Insufficient evidence indicates preliminary or hypothesis-generating findings with substantial uncertainty. ^b^ REDs, Relative Energy Deficiency in Sport; LEA, low energy availability; EPA, eicosapentaenoic acid; DHA, docosahexaenoic acid. Practical considerations should be interpreted as evidence-informed recommendations rather than formal clinical guidelines and should be individualized according to injury characteristics, rehabilitation phase, training load, medical history, dietary intake, and clinical judgment.

**Table 2:** Phase-specific nutrition priorities across tendon/ligament rehabilitation.

Rehabilitation Phase	Primary Goal	Foundation (Do First)	Targeted Strategies (Adjuncts)
Acute/Early	Support early repair environment; avoid low energy availability	Energy sufficiency + adequate protein	Micronutrient adequacy (food-first); avoid “inflammation suppression” framing
Mid/Strength rebuilding	Support ECM remodeling while rebuilding capacity	Energy/protein maintained; distribute protein across day	Collagen/gelatin + vitamin C “pre-loading” before key tendon-loading sessions; creatine monohydrate (3–5 g/day) if disuse-related muscle loss is a concern
Late rehabilitation/Return-to-activity or return-to-sport	Maximize load tolerance; support high-quality training and tissue remodeling	Energy matched to rising load; carbohydrate supports session quality	Omega-3 as adjunct for inflammation resolution; status-based vitamin D/calcium correction

Note: ECM, extracellular matrix. Targeted strategies should be interpreted as adjuncts to foundational nutritional priorities and progressive rehabilitation loading, rather than as standalone interventions.

## Data Availability

No new data were created or analyzed in this study. Data sharing is not applicable to this article.
